# Supplementary material for: Machine learning uncovers cell identity regulator by histone code
Source: Nat Commun. 2020 Jun 1;11:2696. doi: 10.1038/s41467-020-16539-4 (PMC7264183; doi:10.1038/s41467-020-16539-4)
Supplement: Supplementary file 1 — Supplementary Information [file 41467_2020_16539_MOESM1_ESM.pdf]

SUPPLEMENTARY INFORMATION

Machine Learning uncovers cell identity regulator by histone code

Bo Xia[1, 2, 3, 4, #], Dongyu Zhao[1, 2, 3, 4, #], Guangyu Wang[1, 2, 3, 4, #], Min Zhang[2, 3, 4], Jie Lv[1, 2, 3, 4], Alin S. Tomoiaga[5], Yanqiang Li[1, 2, 3, 4], Xin Wang[1, 2, 3, 4], Shu Meng[2, 3, 4], John P. Cooke[2, 3, 4], Qi Cao[6*], Lili Zhang[2, 3, 4, *], Kaifu Chen[1, 2, 3, 4, 7*]

Supplementary Figures 1-17

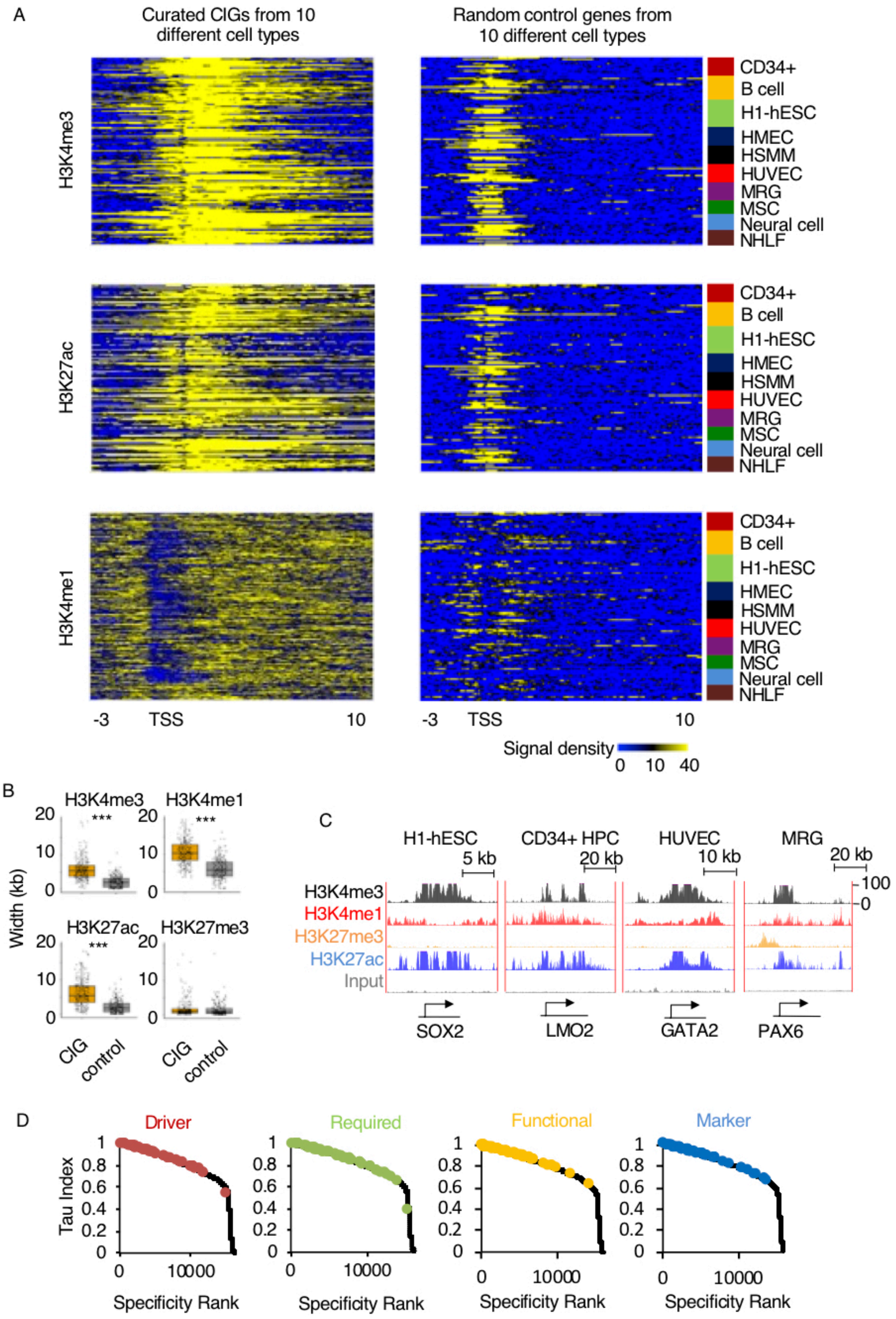

Supplementary Figure 1. Manually curated CIGs for 10 important cell types appear different from random control genes in their associated epigenetic signatures. **(A)** Epigenetic profiles of curated CIGs from

10 different cell types. Heatmap to show signal density for H3K4me3, H3K27ac and H3Kme1 at each base pair (columns) around transcription start sites (rows) of the curated identity genes (left) and control genes (right) for 10 different cell types. (**B**) Box plots to show difference in width of histone modification between CIGs and random control genes. P values determined by Wilcoxon tests, ***, $P < 1 \times 10^{-30}$. Centre line is median, boxes show first and third quartiles, whiskers extend to the most extreme data points that are no more than 1.5 fold of the interquartile range from the box. (**C**) Histone modification signals at 4 example CIGs loci, with the associated cell types indicated on top and gene names indicated at the bottom. (D) Tau index of expression specificity (y-axis) for individual genes ranked on the x-axis. Black color indicate all genes, with each category of CIGs highlighted by the other colors.

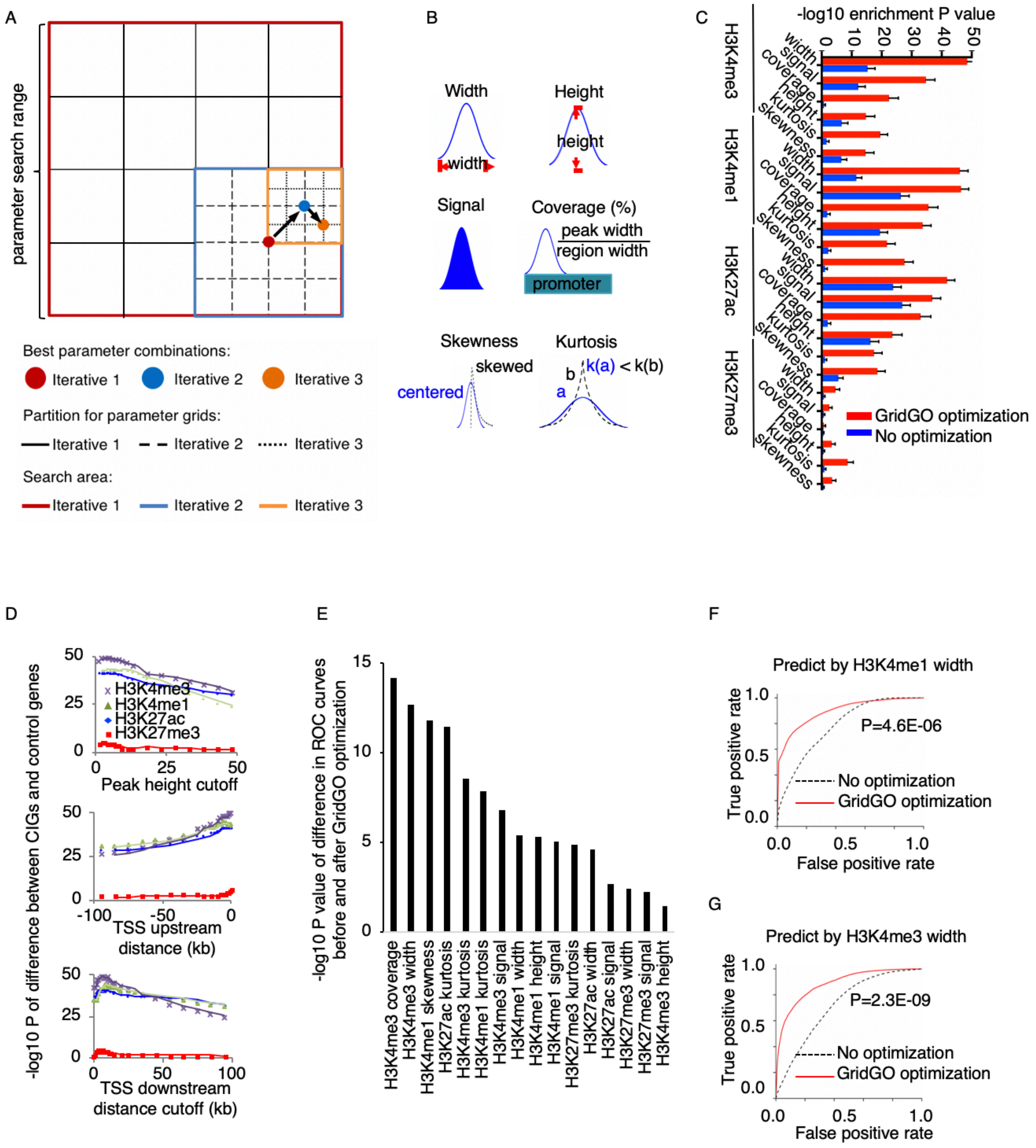

Supplementary Figure 2. The Grid-GO algorithm developed to automatically refine the parameters for detection of epigenetic signature at CIGs. (**A**) The workflow in GridGO. (**B**) Cartoons to show the definition of each feature for ChIP-Seq enrichment peak. (**C**) Bar plots to show -log10 P value of difference in each epigenetic signature between CIGs and random control genes. (**D**) -log10 P values of difference in width of enrichment peak for each histone modification between CIGs and control genes plotted as a function of the ChIP-Seq reads density cutoff to call peak (**top**), the upstream distance cutoff to assign a peak to transcription start site (**middle**), or the downstream distance cutoff to assign a peak to transcription start site (**bottom**). (**E**) Bar plots to show -log10 P values of improvement on the ROC for CIG prediction based on each epigenetic signature after GridGO optimization. (**F-G**) ROC curves to show the accuracy for predicting CIGs by width of enrichment peak defined for H3K4me1 (**F**) or H3K4me3 (**G**) before and after GridGO optimization.

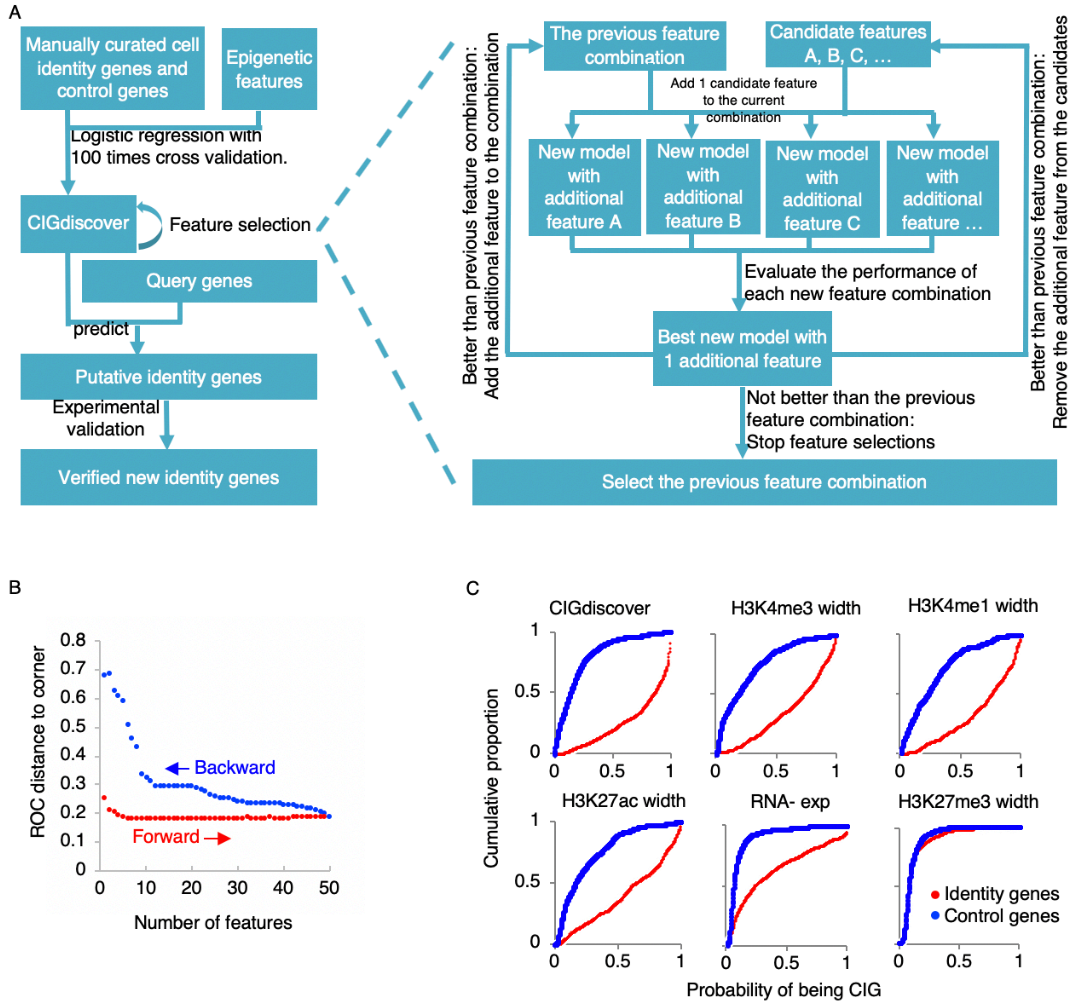

Supplementary Figure 3. The CIGdiscover algorithm developed for cell identity genes discovery. (**A**) The workflow for cell identity gene discovery based on CIGdiscover. (**B**) Distance between ROC curve and top left corner plotted against the number of epigenetic features selected for CIGdiscover by the forward feature construction or backward feature elimination method. **(C)** Cumulative proportion plotted against probability of being CIGs predicted using CIGdiscover, H3K4me3, H3K4me1, H3K27ac, H3K27me3 and RNA expression level.

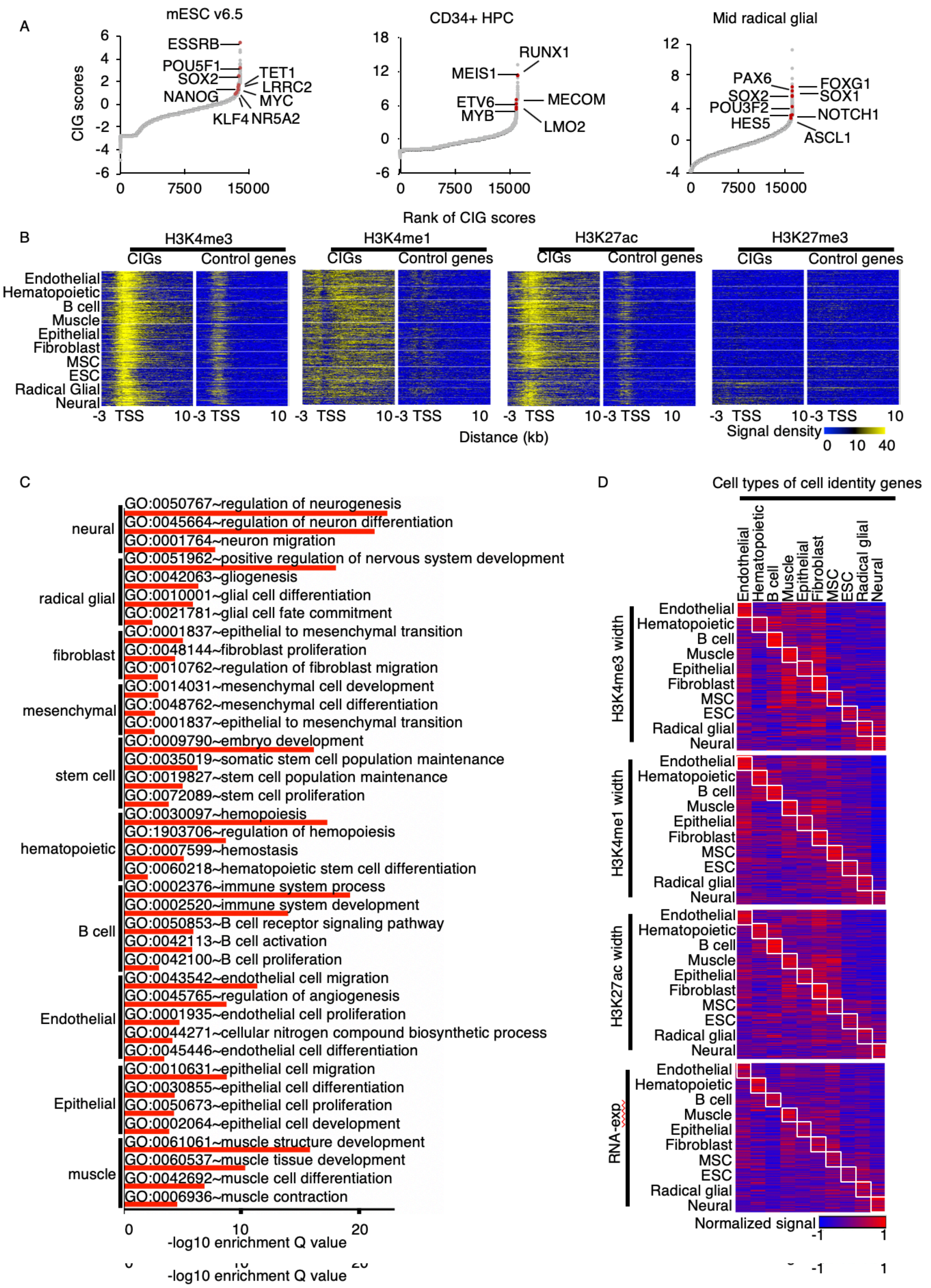

Supplementary Figure 4. CIGs defined by CIGdiscover for the 10 training cell types. **(A)** CIG score defined by CIGdiscover for each gene plotted against rank of CIG score in mESC (left), HPC (middle), and glial (right) cells. Examples of known CIG are marked in red color. **(B)** Heatmap to show signal density for H3K4me3, H3K4me1, H3K27ac and H3K27me3 at each base pair (columns) around transcription start sites (rows) of the uncovered identity genes and control genes for 10 different cell types. (**C**) Bar plots to show -log10 enrichment P values of individual pathways in CIGs defined by CIGdiscover for each cell type. (**D**) Heatmap to show H3K4me3, H3K4me1, H3K27ac peak width and expression level for the uncovered CIGs across 10 different cell types.

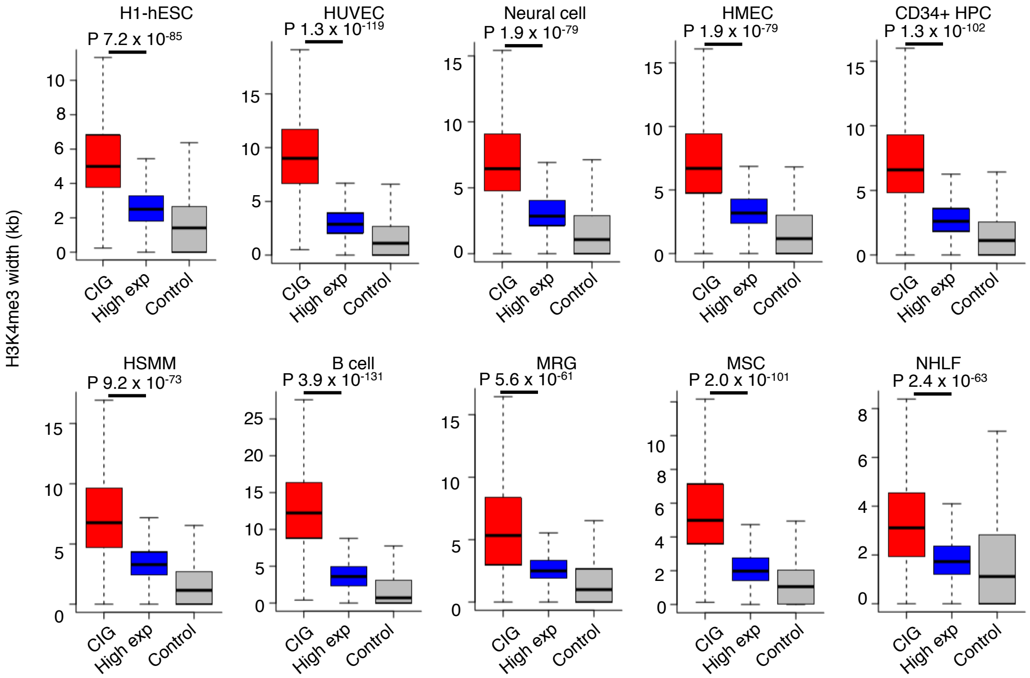

Supplementary Figure 5. H3K4me3 width at putative CIGs defined by CIGdiscover, by high expression, and by random selection. P values were determined by Wilcoxon tests. Box plots: centre line is median, boxes show first and third quartiles, whiskers extend to the most extreme data points that are no more than 1.5 fold of the interquartile range from the box.

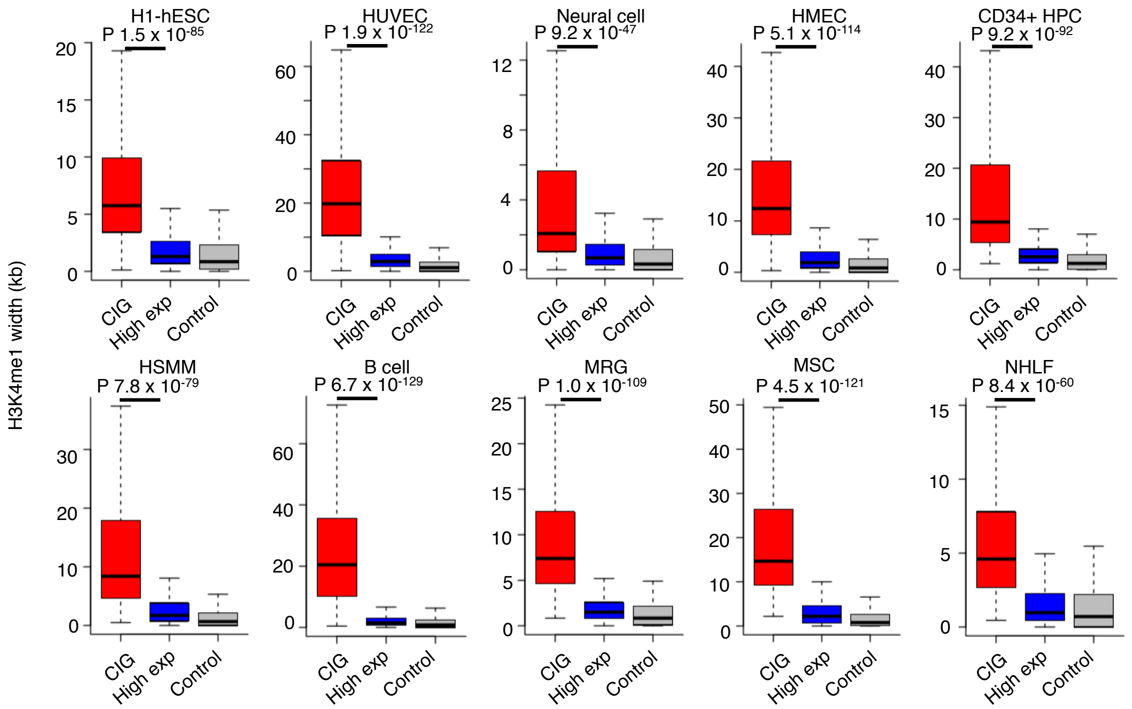

Supplementary Figure 6. H3K4me1 width at putative CIGs defined by CIGdiscover, by high expression, and by random selection. P values were determined by Wilcoxon tests. Box plots: centre line is median, boxes show first and third quartiles, whiskers extend to the most extreme data points that are no more than 1.5 fold of the interquartile range from the box.

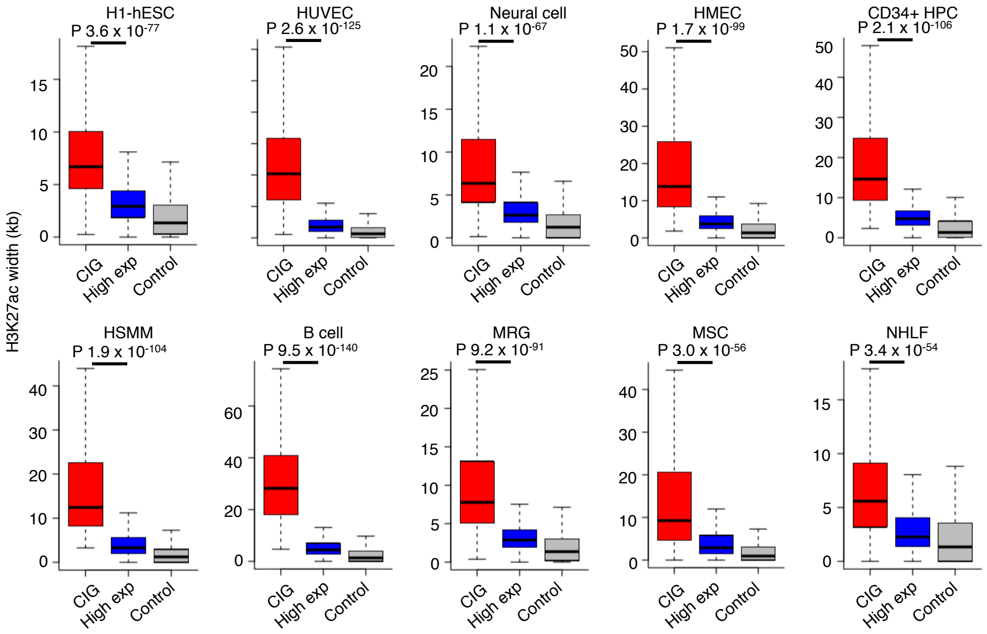

Supplementary Figure 7. H3K27ac width at putative CIGs defined by CIGdiscover, by high expression, and by random selection. P values were determined by Wilcoxon tests. Box plots: centre line is median, boxes show first and third quartiles, whiskers extend to the most extreme data points that are no more than 1.5 fold of the interquartile range from the box.

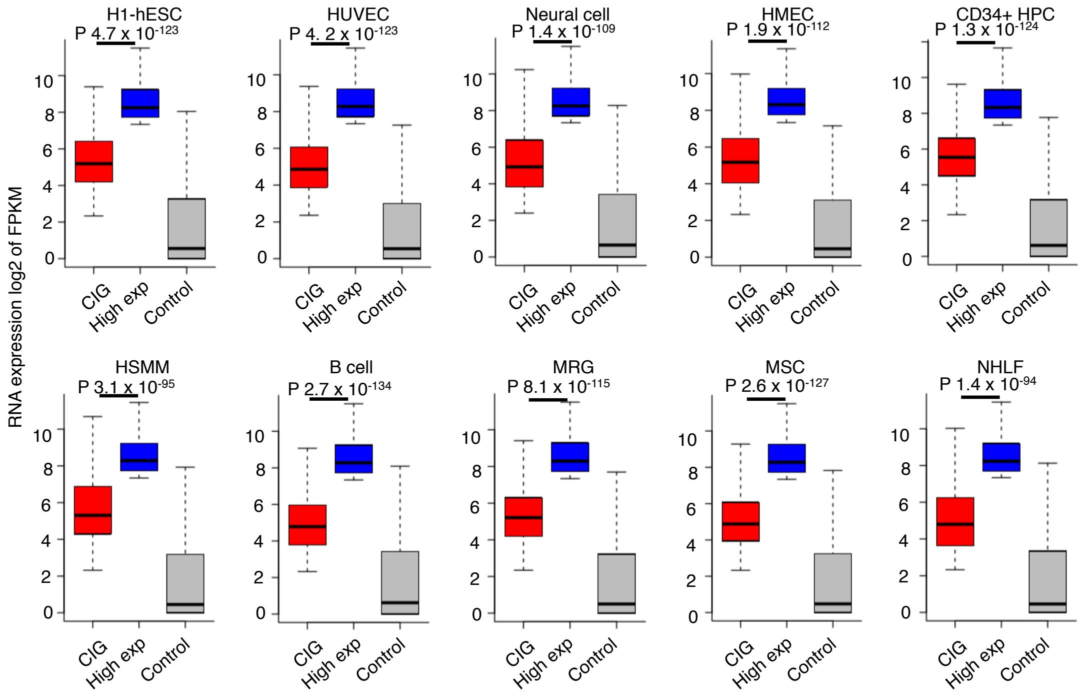

Supplementary Figure 8. RNA expression of putative CIGs defined by CIGdiscover, by high expression, and by random selection. P values were determined by Wilcoxon tests. Box plots: centre line is median, boxes show first and third quartiles, whiskers extend to the most extreme data points that are no more than 1.5 fold of the interquartile range from the box.

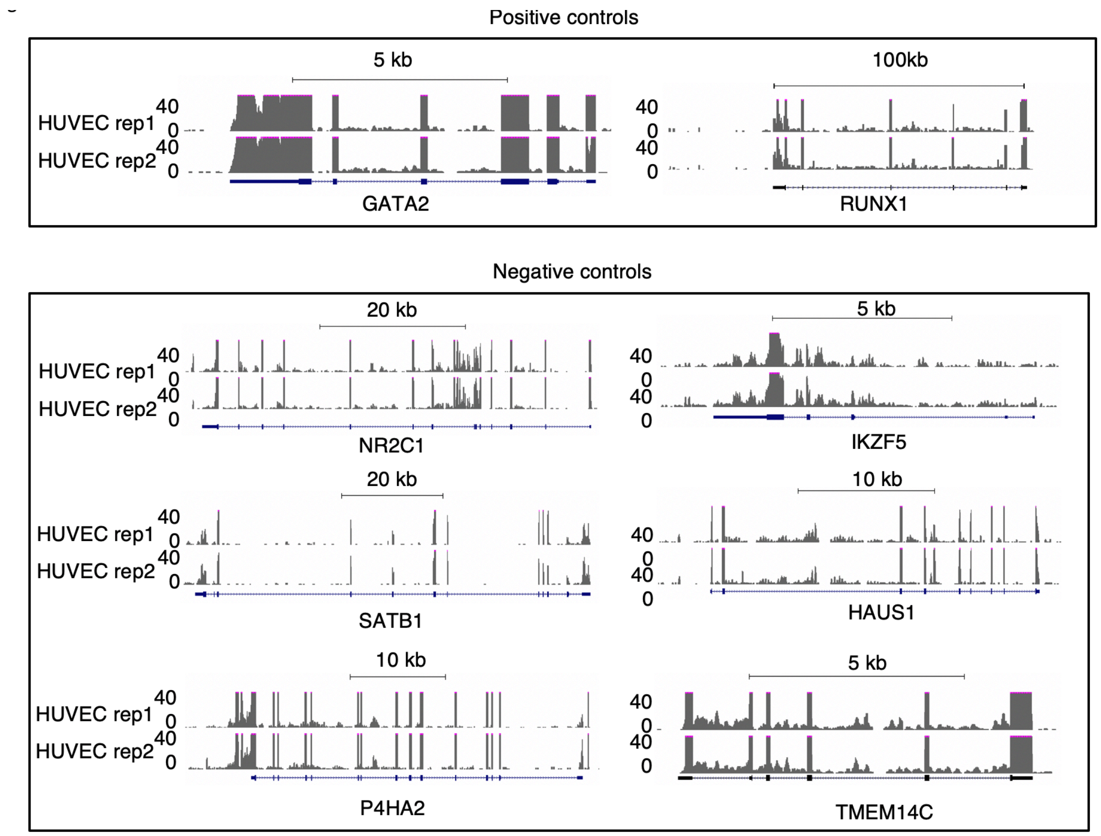

Supplementary Figure 9. UCSC genome browser tracks to show expression of individual positive and negative controls genes in HUVECs.

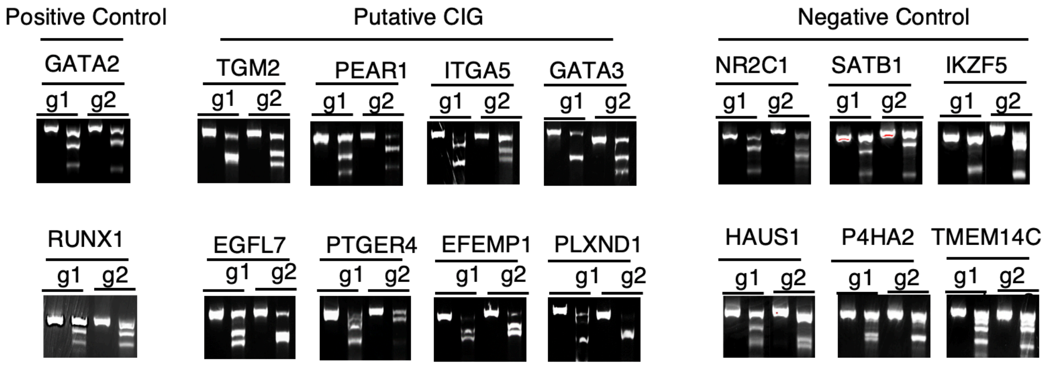

Supplementary Figure 10. T7 Endonuclease cleavage assay to confirm cutting efficiency of the CRISPR-Cas9 system at individual genes in HUVECs. Two guide RNAs g1 and g2 were used for each gene. For each guide RNA, the left and right lanes indicate results from wild type control cells with empty vectors and from knockout cells, respectively. Source data are provided as a Source Data file.

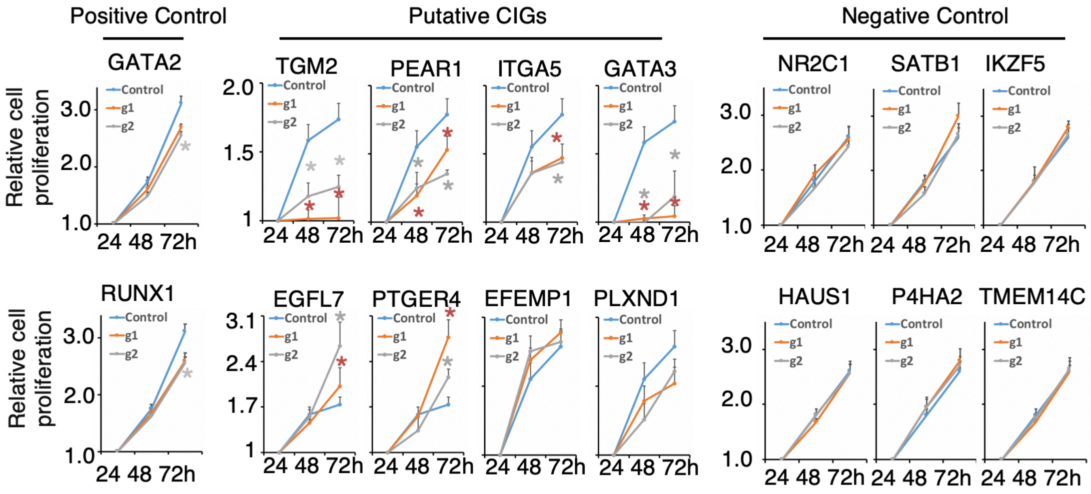

Supplementary Figure 11. Experimental validation of the effects of predicted CIGs on endothelial cell proliferation. Proliferation rate (Y axis) of HUVECs before or after disruption of each endothelial CIG using CRISPR-Cas9 system was plotted at 3 time points (X axis). Two CRISPR-Cas9 gRNAs g1 and g2 were tested. Experiments were repeated 6 times for each condition. P values determined by Student's T test. *, P <0.05. Source data are provided as a Source Data file.

Figure S12

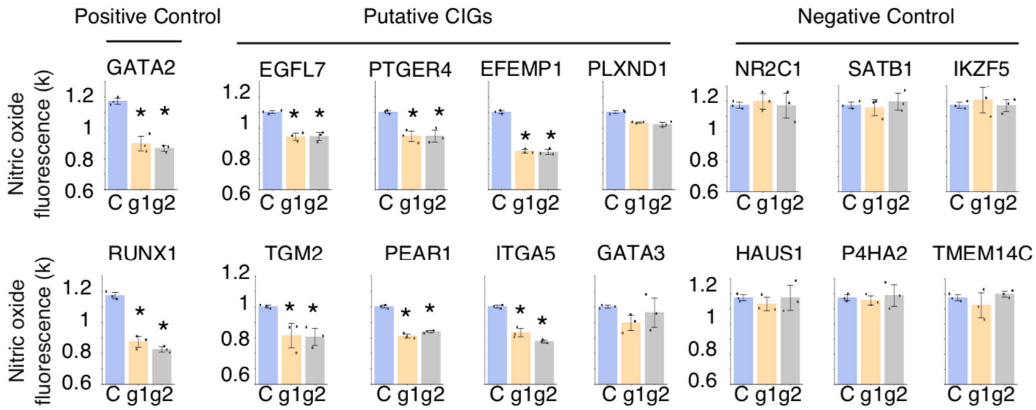

Supplementary Figure 12. Experimental validation of the effects of predicted CIGs on endothelial cell nitric oxide production. Nitric oxide production (Y axis) of HUVECs before or after disruption of each endothelial CIG using CRISPR-Cas9 system was plotted. Two CRISPR-Cas9 gRNAs g1 and g2 were tested. P values determined by Student's T test. *, P <0.05. Source data are provided as a Source Data file.

Figure S13

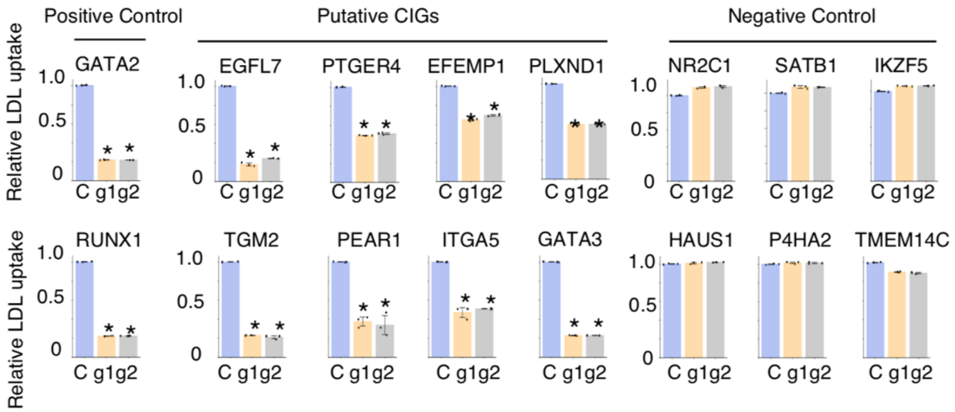

Supplementary Figure 13. Experimental validation of the effects of predicted CIGs on endothelial cell LDL uptake. LDL uptake of HUVECs before or after disruption of each endothelial CIG using CRISPR-Cas9 system was plotted. Two CRISPR-Cas9 gRNAs g1 and g2 were tested. Experiments were repeated 6 times for each condition. P values determined by Student's T test. *, P <0.05. Source data are provided as a Source Data file.

Figure S14

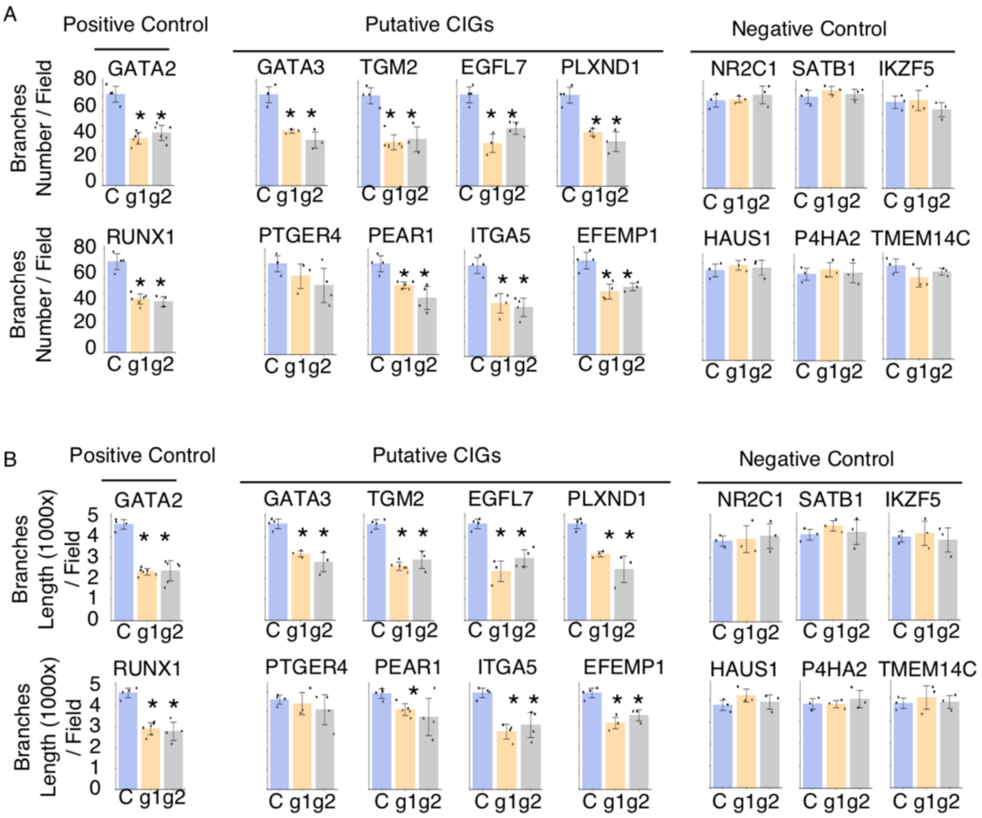

Supplementary Figure 14. Experimental validation of the effects of predicted CIGs on endothelial cell tube formation. Tube branch number (**A**) and length (**B**) of HUVEC before or after disruption of each endothelial CIG using CRISPR-Cas9 system were plotted. Two CRISPR-Cas9 gRNAs g1 and g2 were tested. Experiments were repeated 6 times for each condition. P values determined by Student's T test. *, $P < 0.05$. Source data are provided as a Source Data file.

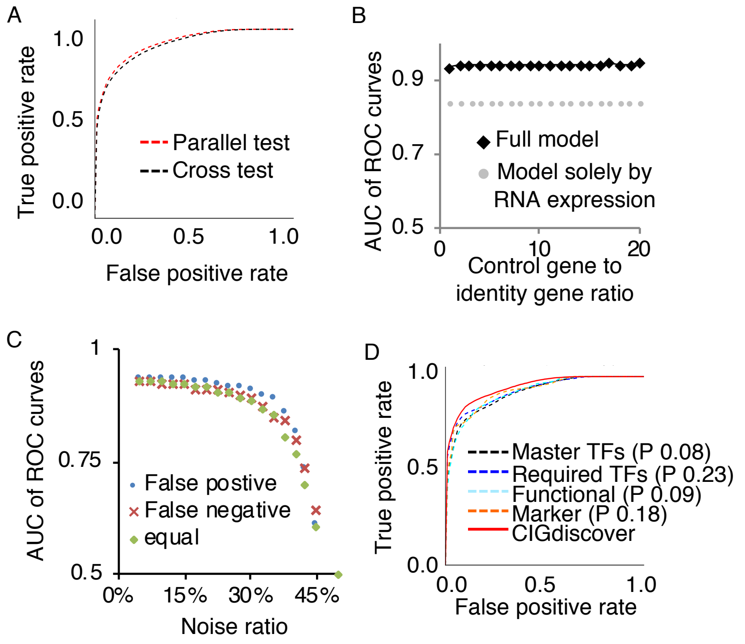

Figure S15. CIGdiscover is robust and resistant to noise or small training dataset. (**A**) ROC curves to show the performances of CIGdiscover when trained and tested by data from the same set (parallel test) or two different sets (cross test) of cell types. (**B**) AUC of ROC plotted against control gene to CIG ratio in the training data for CIGdiscover. We increase the ratio by increasing the number of control genes while matain the number of CIGs. (**C**) AUC of ROC plotted against noise ratio in the training data for CIGdiscover. False positive: only swap negative control genes to identity genes; False negative: only swap identity genes to negative control genes; equal: swap equal number of identity genes to control genes and control genes to identity genes. (**D**) ROC curves to show performance of CIGdiscover variants trained by all categories or each single category of CIGs but tested by all categories.

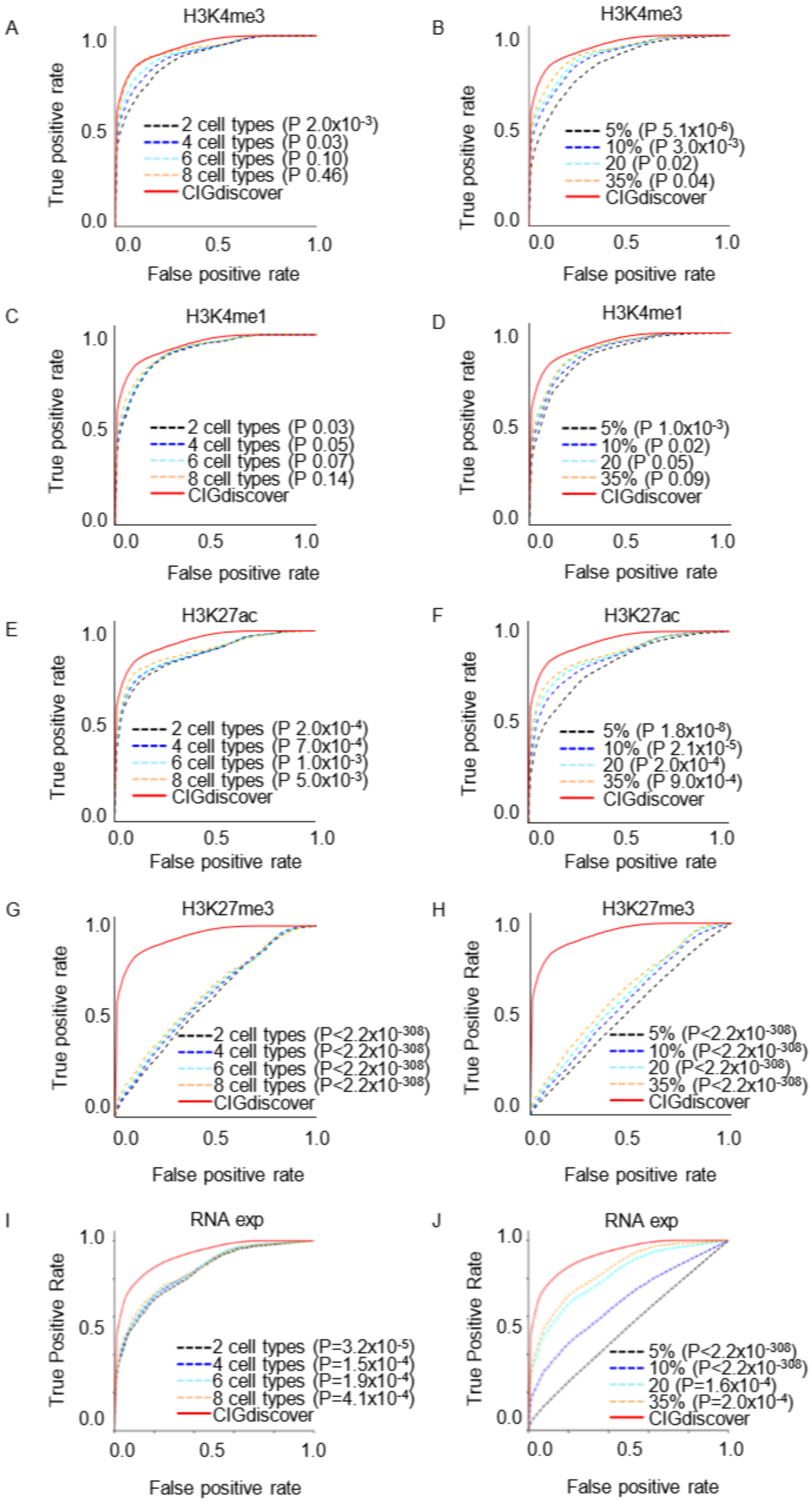
A
H3K4me3
True positive rate
False positive rate
2 cell types (P 2.0x10-3)
4 cell types (P 0.03)
6 cell types (P 0.10)
8 cell types (P 0.46)
CIGdiscover
B
H3K4me3
5% (P 5.1x10-6)
10% (P 3.0x10-3)
20 (P 0.02)
35% (P 0.04)
CIGdiscover
C
H3K4me1
2 cell types (P 0.03)
4 cell types (P 0.05)
6 cell types (P 0.07)
8 cell types (P 0.14)
CIGdiscover
D
H3K4me1
5% (P 1.0x10-3)
10% (P 0.02)
20 (P 0.05)
35% (P 0.09)
CIGdiscover
E
H3K27ac
2 cell types (P 2.0x10-4)
4 cell types (P 7.0x10-4)
6 cell types (P 1.0x10-3)
8 cell types (P 5.0x10-3)
CIGdiscover
F
H3K27ac
5% (P 1.8x10-8)
10% (P 2.1x10-5)
20 (P 2.0x10-4)
35% (P 9.0x10-4)
CIGdiscover
G
H3K27me3
2 cell types (P<2.2x10-308)
4 cell types (P<2.2x10-308)
6 cell types (P<2.2x10-308)
8 cell types (P<2.2x10-308)
CIGdiscover
H
H3K27me3
True Positive Rate
5% (P<2.2x10-308)
10% (P<2.2x10-308)
20 (P<2.2x10-308)
35% (P<2.2x10-308)
CIGdiscover
I
RNA exp
True Positive Rate
2 cell types (P=3.2x10-5)
4 cell types (P=1.5x10-4)
6 cell types (P=1.9x10-4)
8 cell types (P=4.1x10-4)
CIGdiscover
J
RNA exp
5% (P<2.2x10-308)
10% (P<2.2x10-308)
20 (P=1.6x10-4)
35% (P=2.0x10-4)
CIGdiscover
0.0
0.5
1.0

Supplementary Figure 16. ROC curves to show performance of individual CIGdiscover variants. The variants in different panels utilized only H3K4me3 (**A, B**), H3K4me1 (**C, D**), H3K27ac (**E, F**), or H3K27me3 (**G, H**), or RNA expression (**I, J**). The different variants presented within each panel were trained by reported CIGs from different number of cell types (**A, C, E, G, I**) or were trained by different subsets of reported CIGs (**B, D, F, H, J**).

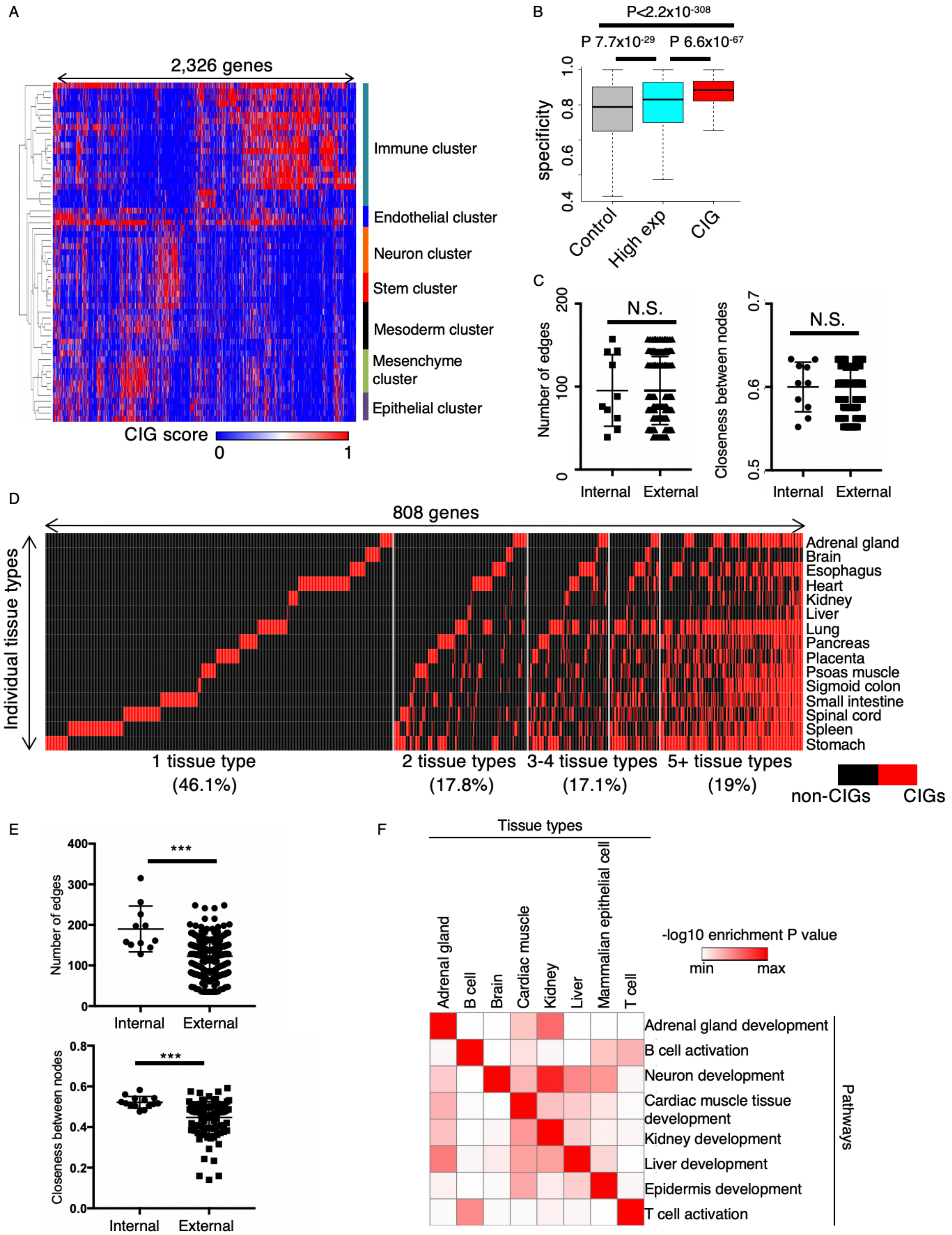

Supplementary Figure 17. A comprehensive landscape of CIGs uncovered by CEFCIG. (**A**) Heatmap and hierarchy clustering analysis for CIGs uncovered for each of 57 cell types. (**B**) Boxplot to show expression specificity of CIGs, high expression genes, and random control genes. Centre line is median, boxes show first and third quartiles, whiskers extend to the most extreme data points that are no more than 1.5 fold of

the interquartile range from the box (**C**) One-dimensional scatter plots to show number of network edges and closeness between high expression genes from the same cell type (internal) or from different cell types (external). (**D**) Heatmap to show tissue identity genes uncovered by CEFCIG for 15 tissue types. (**E**) One-dimensional scatter plots to show number of network edges (**top**) and closeness (**bottom**) between identity genes from the same (internal) or different tissue types (external). P values are calculated by Wilcoxon test *** $P<0.001$. (**F**) Heatmap to show -log10 enrichment P values of tissue-related pathways in identity genes of distinct tissue types.
