# Supplementary material for: Machine learning uncovers cell identity regulator by histone code
Source: Nat Commun. 2020 Jun 1;11:2696. doi: 10.1038/s41467-020-16539-4 (PMC7264183; doi:10.1038/s41467-020-16539-4)
Supplement: Supplementary file 3 — Description of Additional Supplementary Files [file 41467_2020_16539_MOESM3_ESM.pdf]

Description of Additional Supplementary Files

File Name: Supplementary Data 1
Description: Curated cell identity genes

File Name: Supplementary Data 2
Description: Predicted endothelial cells cell identity genes

File Name: Supplementary Data 3
Description: Datasets used in training, test, and landscape analysis

File Name: Supplementary Data 4
Description: Primer Design
