# Supplementary material for: Machine learning uncovers cell identity regulator by histone code
Source: Nat Commun. 2020 Jun 1;11:2696. doi: 10.1038/s41467-020-16539-4 (PMC7264183; doi:10.1038/s41467-020-16539-4)
Supplement: Supplementary file 8 — Reporting Summary [file 41467_2020_16539_MOESM8_ESM.pdf]

nature research

Corresponding author(s): *Double-blind peer review submissions: write DBPR and your manuscript number here instead of author names.*

Last updated by author(s): *YYYY-MM-DD*

Reporting Summary

Nature Research wishes to improve the reproducibility of the work that we publish. This form provides structure for consistency and transparency in reporting. For further information on Nature Research policies, see Authors & Referees and the Editorial Policy Checklist.

Statistics

For all statistical analyses, confirm that the following items are present in the figure legend, table legend, main text, or Methods section.

n/a	Confirmed	
☐	☒	The exact sample size (n) for each experimental group/condition, given as a discrete number and unit of measurement
☐	☒	A statement on whether measurements were taken from distinct samples or whether the same sample was measured repeatedly
☐	☒	The statistical test(s) used AND whether they are one- or two-sided<br>*Only common tests should be described solely by name; describe more complex techniques in the Methods section.*
☒	☐	A description of all covariates tested
☐	☒	A description of any assumptions or corrections, such as tests of normality and adjustment for multiple comparisons
☐	☒	A full description of the statistical parameters including central tendency (e.g. means) or other basic estimates (e.g. regression coefficient) AND variation (e.g. standard deviation) or associated estimates of uncertainty (e.g. confidence intervals)
☒	☐	For null hypothesis testing, the test statistic (e.g. F, t, r) with confidence intervals, effect sizes, degrees of freedom and P value noted<br>*Give P values as exact values whenever suitable.*
☒	☐	For Bayesian analysis, information on the choice of priors and Markov chain Monte Carlo settings
☒	☐	For hierarchical and complex designs, identification of the appropriate level for tests and full reporting of outcomes
☒	☐	Estimates of effect sizes (e.g. Cohen's d, Pearson's r), indicating how they were calculated

Our web collection on statistics for biologists contains articles on many of the points above.

Software and code

Policy information about availability of computer code

Data collection	All RNAseq, ChipSeq data are collected from GEO and ENCODE with accession ID. Cell identity genes are curated with PMID annotated in Table S1.
Data analysis	All custom codes are developed by Python with numpy, scipy, pandas, sklearn, matplotlib. Statistical test is performed by using function within scipy or R. All codes are deposited into "https://github.com/bxia888/CEFCIG".

For manuscripts utilizing custom algorithms or software that are central to the research but not yet described in published literature, software must be made available to editors/reviewers. We strongly encourage code deposition in a community repository (e.g. GitHub). See the Nature Research guidelines for submitting code & software for further information.

Data

Policy information about availability of data

All manuscripts must include a data availability statement. This statement should provide the following information, where applicable:

- Accession codes, unique identifiers, or web links for publicly available datasets
- A list of figures that have associated raw data
- A description of any restrictions on data availability

N/A

Field-specific reporting

Please select the one below that is the best fit for your research. If you are not sure, read the appropriate sections before making your selection.

☒ Life sciences ☐ Behavioural & social sciences ☐ Ecological, evolutionary & environmental sciences

For a reference copy of the document with all sections, see nature.com/documents/nr-reporting-summary-flat.pdf

Life sciences study design

All studies must disclose on these points even when the disclosure is negative.

Sample size	All the experiment were performed in cell line. 3-6 individual samples were performed in each group
Data exclusions	No data were excluded from the analysis
Replication	All experiments have been repeated for at least 3 times. All experiments were reproduced to reliably support conclusions stated in the manuscript.
Randomization	Cells in different group were passaged from the same cell line and randomly assigned to experimental groups. All experiments' results from individual samples are reported.
Blinding	After cell treatment, different groups were labeled with numbers only. During the data collection and analysis, the investigators were blinded to group allocation.

Reporting for specific materials, systems and methods

We require information from authors about some types of materials, experimental systems and methods used in many studies. Here, indicate whether each material, system or method listed is relevant to your study. If you are not sure if a list item applies to your research, read the appropriate section before selecting a response.

Materials & experimental systems

n/a	Involved in the study	
☐	☒	Antibodies
☐	☒	Eukaryotic cell lines
☒	☐	Palaeontology
☒	☐	Animals and other organisms
☒	☐	Human research participants
☒	☐	Clinical data

Methods

n/a	Involved in the study	
☒	☐	ChIP-seq
☐	☒	Flow cytometry
☒	☐	MRI-based neuroimaging

Antibodies

Antibodies used	All FACS antibodies are commercially available and have been tested for the species used in this manuscript. Following antibodies were used:<br>1. CD31 (PECAM-1) Monoclonal Antibody (WM-59 (WM59)), APC, eBioscience™, Catalog # 17-0319-41, Thermo Scientific<br>2. CD144 (VE-cadherin) Monoclonal Antibody (16B1), Alexa Fluor 488, eBioscience™, Catalog # 53-1449-41, Thermo Scientific
Validation	Those antibodies were validated by Thermo Fisher Scientific, and the validation data were listed on their antibody website. 14 published Figures and citations are available for those antibodies.

Eukaryotic cell lines

Policy information about cell lines

Cell line source(s)	Human Umbilical Vein Endothelial Cells (HUVEC) and hPSC (Human pluripotent stem cells) were purchased from ATCC
Authentication	All the cell line authentication were performed by ATCC
Mycoplasma contamination	All the cell line tested negative for Mycoplasma contamination
Commonly misidentified lines (See ICLAC register)	N/A

Flow Cytometry

Plots

Confirm that:

☒ The axis labels state the marker and fluorochrome used (e.g. CD4-FITC).

☒ The axis scales are clearly visible. Include numbers along axes only for bottom left plot of group (a 'group' is an analysis of identical markers).

☐ All plots are contour plots with outliers or pseudocolor plots.

☒ A numerical value for number of cells or percentage (with statistics) is provided.

Methodology

Sample preparation	10 days after differentiation, iPSCs were trypsinised, centrifuged at 200 x g for 5min, resuspended in FACSB-10 (FACS buffer–10% FBS) and incubated with anti human-VE cadherin and anti human CD31 for 30 min on ice
Instrument	Fluorescence was determined using a flow cytometer (LSR II, Becton Dickinson, San Jose, CA, USA)
Software	The data were analyzed using FlowJo software.
Cell population abundance	Purity of post-sort fractions is regularly measured by flow cytometry core facility and >90%
Gating strategy	Single cells are identified by plotting forward scatter-area against forward scatter-height. single cells are then separated from debris by a forward versus side scatter. CD31+CD144+ cells were defined as induced endothelial cells.

☐ Tick this box to confirm that a figure exemplifying the gating strategy is provided in the Supplementary Information.
